# Racial/Ethnic, Sex, and Economic Disparities in the Utilization and Outcomes of Intracoronary Imaging

**DOI:** 10.1016/j.jscai.2024.101936

**Published:** 2024-05-11

**Authors:** Mahmoud Ismayl, Hasaan Ahmed, Andrew M. Goldsweig, Mohamad Alkhouli, Abhiram Prasad, Mayra Guerrero

**Affiliations:** a Department of Cardiovascular Medicine, Mayo Clinic, Rochester, Minnesota; b Department of Internal Medicine, Creighton University School of Medicine, Omaha, Nebraska; c Department of Cardiovascular Medicine, Baystate Medical Center, Springfield, Massachusetts

**Keywords:** economic disparities, intracoronary imaging, intravascular ultrasound, percutaneous coronary angiography, racial disparities, sex disparities

## Abstract

**Background:**

Intracoronary imaging–guided percutaneous coronary intervention (PCI) is associated with improved outcomes compared with angiography–guided PCI. Data on racial/ethnic, sex, and economic disparities in the utilization and outcomes of intracoronary imaging in the United States are scarce.

**Methods:**

We analyzed the National Inpatient Sample (2016-2020) to examine racial/ethnic, sex, and economic differences in the utilization of intracoronary imaging among patients who underwent PCI. Trends, in-hospital mortality, and safety of intracoronary imaging were also assessed.

**Results:**

Among 2,212,595 weighted hospitalizations for PCI, 204,735 (9.2%) included intracoronary imaging. The utilization rate of intracoronary imaging was similar in Black and Hispanic patients compared with White patients (9.8% vs 10.2% vs 10.0%; *P* = .68) and lower for women compared with men (10.0% vs 10.3%; *P* = .01) and for patients with low and medium income compared with high income (9.2% vs 10.0% vs 12.5%; *P* < .01). In multivariable regression analysis, low and medium income were independently associated with lower intracoronary imaging use compared with high income (both *P* < .01). From 2016 through 2020, the use of intracoronary imaging in PCI increased significantly in all racial/ethnic, sex, and economic groups (all *P*_trend_ < .01). Among patients who underwent PCI with intracoronary imaging, Black race was associated with higher odds of acute kidney injury compared with White race (adjusted odds ratio, 1.40; 95% CI, 1.25-1.57). In-hospital mortality was similar between different racial/ethnic, sex, and economic groups.

**Conclusions:**

Low and medium income are independently associated with lower intracoronary imaging use in PCI compared with high income. Further studies are needed to identify effective strategies to mitigate economic disparities in intracoronary imaging use.

## Introduction

Coronary artery disease remains the most prevalent cause of mortality globally, with coronary angiography considered the gold standard for diagnosis and assessing the need for revascularization by percutaneous coronary intervention (PCI) or coronary artery bypass grafting.^1^^,^^2^ Limitations of coronary angiography, evident by its 2-dimensional imaging with its inability to evaluate internal arterial dimensions and plaque composition fully, have led to increased utilization of intracoronary imaging with intravascular ultrasound (IVUS) and optical coherence tomography (OCT).^1^^,^^3^ These adjunctive imaging modalities complement coronary angiography to improve outcomes among patients who undergo PCI, with intracoronary imaging-guided PCI associated with decreased rates of mortality, target lesion revascularization, and myocardial infarction compared with coronary angiography alone.^2^^,^^4^^,^^5^

Despite an overall decrease in both the incidence of acute coronary syndrome and mortality from ischemic heart disease, racial/ethnic, sex, and economic disparities persist.^6^^,^^7^ Elevated rates of medical comorbidities among minority groups in combination with socioeconomic and geographic factors reinforce these disparities.^6^^,^^7^ Although there have been significant advancements in optimizing invasive treatment of coronary artery disease, the impact of racial/ethnic, sex, and economic disparities with respect to intracoronary imaging has not yet been examined. Furthermore, the effect of intracoronary imaging on PCI outcomes disparities is unknown. Therefore, we analyzed the National Inpatient Sample (NIS) database to evaluate for racial/ethnic, sex, and economic disparities in the utilization and outcomes of intracoronary imaging among patients undergoing PCI.

## Materials and methods

### Data source and ethics statement

Hospitalization data were abstracted from the NIS database, which is part of the Healthcare Cost and Utilization Project (HCUP) family of databases sponsored by the Agency for Healthcare Research and Quality.^8^ The specific data supporting this study’s findings are available from the corresponding author upon request. The NIS is the largest publicly available, fully deidentified, all-payer inpatient health care database in the United States. The NIS is derived from billing data submitted by hospitals to statewide organizations across the United States and has reliable and verified patient linkage numbers that can be used to track patients across hospitals within each state while adhering to strict privacy guidelines. The NIS database contains both patient-level and hospital-level information from approximately 1000 hospitals and represents approximately 20% of all US hospitalizations, covering >7 million unweighted hospitalizations each year. When weighted, the NIS extrapolates to the national level, representing 35 million hospitalizations each year. Up to 40 discharge diagnoses and 25 procedure codes are collected for each patient using International Classification of Diseases, Tenth Revision (ICD-10) codes.^9^ The NIS is compiled annually, which allows for analysis of procedural trends over time.^10^ This study was exempt from the requirements of the Mayo Clinic institutional review board because the NIS is a publicly available database comprising deidentified data.

### Study population and patient selection

**Figure 1:**
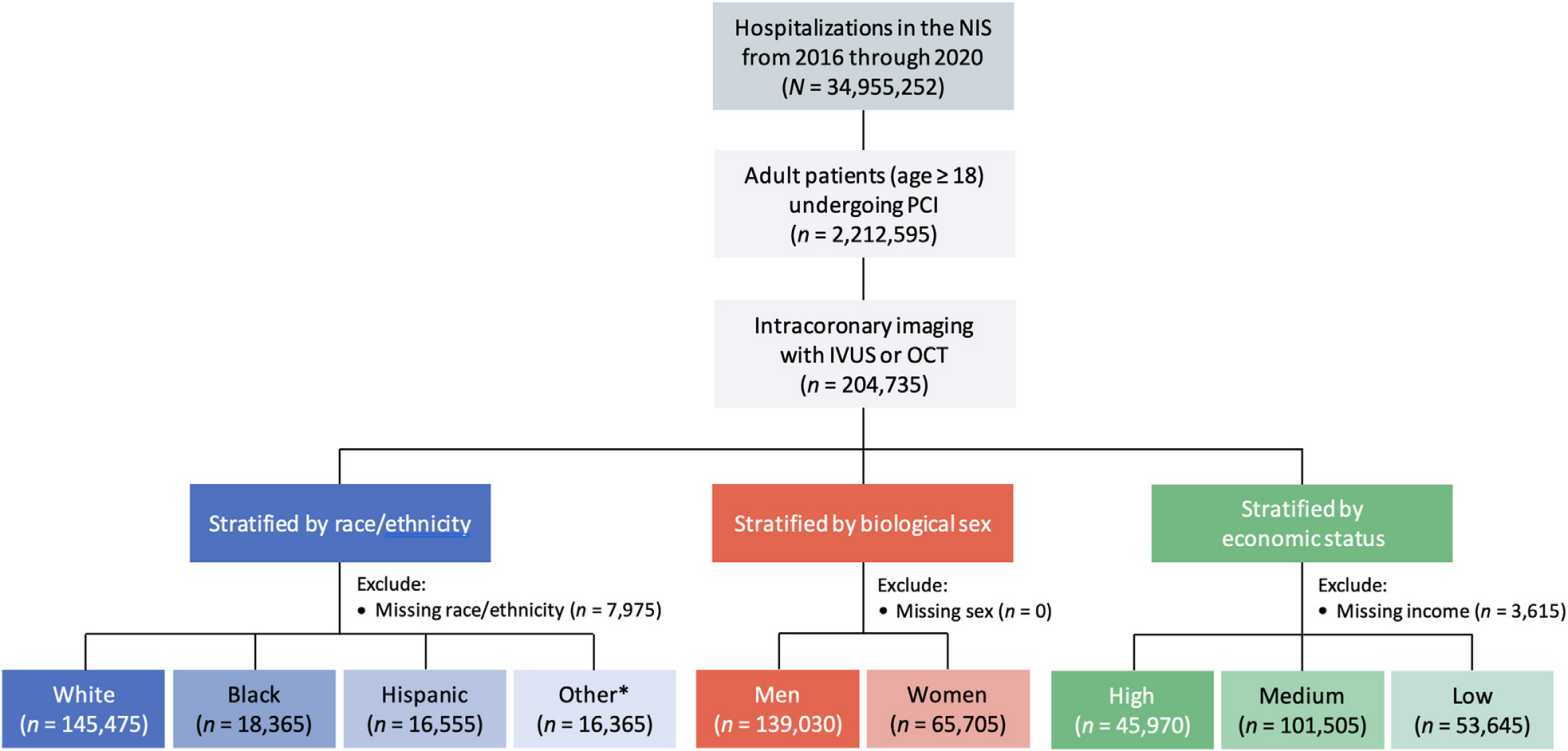
**Study flow diagram showing inclusion and exclusion criteria.** Hospitalization counts represent national-level estimates. ∗Other race refers to Asian or Pacific Islander, Native American, and other. IVUS, intravascular ultrasound; NIS, National Inpatient Sample; OCT, optical coherence tomography; PCI, percutaneous coronary intervention.

We queried the NIS database from January 2016 through December 2020 to identify hospitalizations in which adult patients (aged ≥18 years) underwent PCI and intracoronary imaging with IVUS (ICD-10, Procedure Coding System B240ZZ3, B241ZZ3, B244ZZ3, B245ZZ3, B246ZZ3, and B24DZZ3 in any procedural field) or OCT (ICD-10, Procedure Coding System B221Z2Z and B223Z2Z in any procedural field). A complete list of ICD-10 diagnosis and procedure codes used in this study is presented in Supplemental Table S1. We excluded hospitalizations in which the patients were aged <18 years and those with missing data on race/ethnicity, sex, or income. For hospitalizations that met inclusion criteria, we stratified the total cohort by race/ethnicity (White, Black, Hispanic, and other), biological sex, and economic status (high, medium, and low income) (Figure 1).

NIS combines race and ethnicity into 1 data element (race). If both race and ethnicity were available, HCUP preferred ethnicity over race in assigning a value for the race variable.^11^ Similar to prior NIS studies, 3 racial/ethnic groups with small sample sizes (Asian or Pacific Islander, Native American, and other) were combined into a single other group to facilitate the analysis.^12^^,^^13^ The other 3 HCUP racial/ethnic groups (White, Black, and Hispanic) were left unchanged for the study. “White” group refers to non-Hispanic White patients, “Black” group refers to non-Hispanic Black patients, and “Hispanic” group refers to Hispanic patients of all races and origins.

The estimated median household incomes are ZIP code specific, updated annually, and classified into 4 quartiles indicating the poorest to wealthiest populations. Similar to prior NIS studies, 3 income groups (high, medium, and low) were used to facilitate the analysis.^14^ High income refers to 75th-100th percentile, medium income to 25th to 75th percentile, and low income to 0th to 25th percentile. A more detailed explanation of all the variables in the NIS, including the specific dollar amounts in each category of median household income, is available online (https://www.hcup-us.ahrq.gov/db/nation/nis/nisdde.jsp).

### Study outcomes

The primary outcome was intracoronary imaging utilization rate per race/ethnicity, sex, and economic group. Secondary outcomes included temporal trends in intracoronary imaging use in PCI in different racial/ethnic, sex, and economic groups. In-hospital mortality and safety outcomes following intracoronary imaging including acute kidney injury (AKI) were also assessed. Additional analyses comparing in-hospital mortality and AKI among patients undergoing PCI without intracoronary imaging were also performed.

### Statistical analysis

Descriptive statistics were presented as percentages for categorical variables and as medians with IQRs for continuous variables. Categorical variables were compared using the Pearson χ^2^ test or Fisher exact test as appropriate. Continuous variables were compared using the Mann-Whitney *U* test for comparisons including 2 groups and Kruskal-Wallis 1-way analysis of variance for comparisons including 3 or more groups.

A multivariable logistic regression analysis was constructed to adjust for potential confounders, which included age, race/ethnicity (not included when comparing racial/ethnic groups), sex (not included when comparing sex groups), income (not included when comparing income groups), insurance, hospital location and teaching status, bed size, region, type of admission (elective/nonelective and weekend/weekday), Elixhauser and Charlson comorbidity index scores, and relevant comorbidities (Supplemental Table S2). Adjustment variables were selected a priori on the basis of their clinical significance, which may directly influence in-hospital outcomes. The results from these models are presented as adjusted odds ratio (aOR) with 95% CI. Trend analyses from 2016 through 2020 were conducted using linear regression.

A 2-tailed *P* value of <.05 was considered statistically significant. Given the large sample size, all *P* values that are statistically significant may not be clinically significant and, therefore, need careful clinical interpretation. All statistical analyses were performed using Stata version 17 software, accounting for the NIS sampling design, and were weighted using sampling weights provided with the NIS database to estimate national-level effects per HCUP-NIS recommendations.^10^

## Results

### Utilization rate of intracoronary imaging per race/ethnicity, sex, and economic status

**Figure 2:**
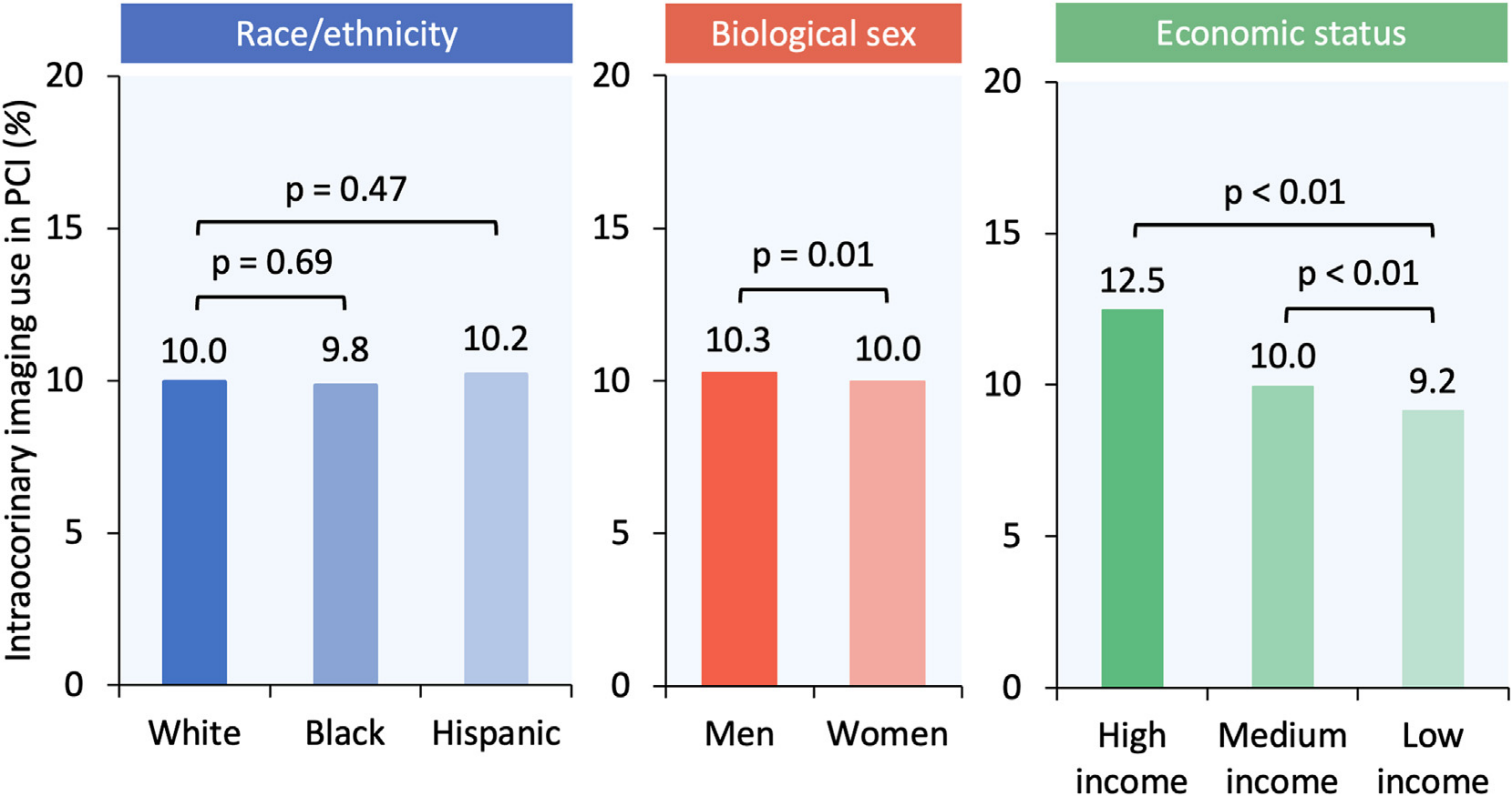
**Racial/ethnic, sex, and economic differences in the utilization of intracoronary imaging in PCI in the United States.** PCI, percutaneous coronary intervention.

Among 2,212,595 weighted hospitalizations for PCI identified in our analysis, 204,735 (9.2%) included intracoronary imaging, of which 145,475 (71.1%) patients were of White race, 139,030 (67.9%) were men, and 45,970 (22.9%) were of high income (Figure 1). The utilization rate of intracoronary imaging in PCI was similar in Black and Hispanic patients compared with White patients (9.8% vs 10.2% vs 10.0%; *P* = .68) (Figure 2) and lower for women compared with men (10.0% vs 10.3%; *P* = .01) (Figure 2) and for patients with low and medium income compared with high income (9.2% vs 10.0% vs 12.5%; *P* < .01) (Figure 2).

**Figure 3:**
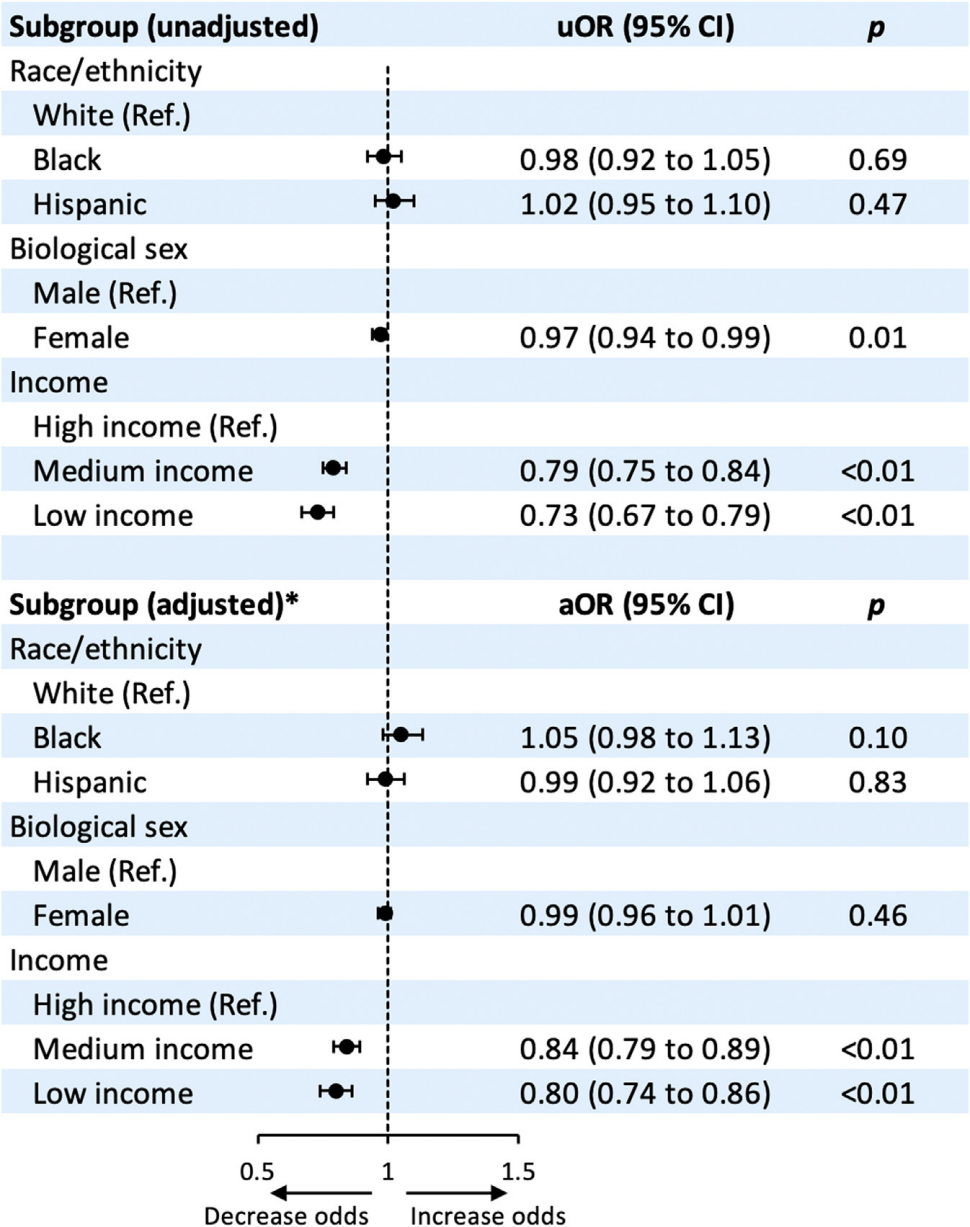
**Forest plot showing variables associated with intracoronary imaging use among patients undergoing PCI.** ∗The multivariable regression model is adjusted for age, race/ethnicity (not included when comparing racial/ethnic groups), sex (not included when comparing sex groups), income (not included when comparing income groups), insurance, hospital location and teaching status, bed size, region, type of admission (elective/nonelective and weekend/weekday), Elixhauser and Charlson comorbidity index scores, and relevant comorbidities. aOR, adjusted odds ratio; PCI, percutaneous coronary intervention; uOR, unadjusted odds ratio.

In multivariable regression analysis, Black race and Hispanic ethnicity compared with White race as well as female sex were not independently associated with lower intracoronary imaging use in PCI (all *P* > .05) (Figure 3). Low and medium income were independently associated with lower intracoronary imaging use in PCI compared with high income (both *P* < .01) (Figure 3). Compared with Medicare insurance, Medicaid insurance was independently associated with lower intracoronary imaging use in PCI (aOR, 0.93; 95% CI, 0.88-0.97; *P* < .01); however, private insurance (aOR, 0.98; 95% CI, 0.94-1.01; *P* = .14) and self-pay (aOR, 0.98; 95% CI, 0.91-1.05; *P* = .52) were not.

### Temporal trends in intracoronary imaging use in PCI

**Figure 4:**
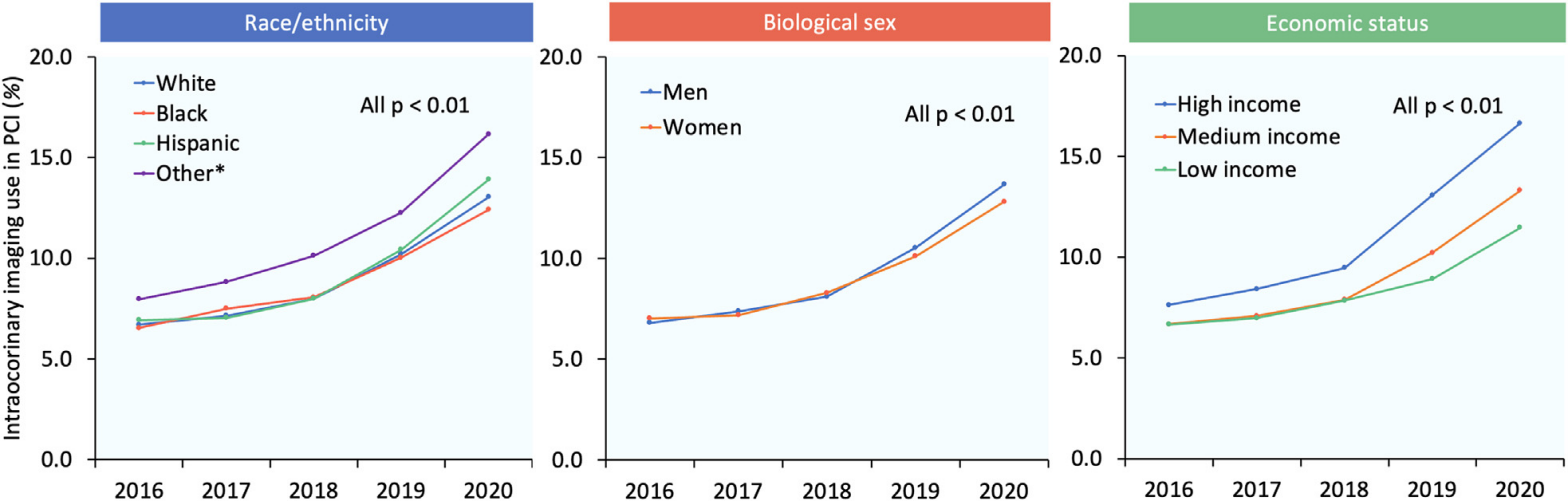
**Year-over-year trend in the use of intracoronary imaging in PCI stratified by race/ethnicity, sex, and economic status.** ∗Other race refers to Asian or Pacific Islander, Native American, and other. PCI, percutaneous coronary intervention.

From 2016 through 2020, the use of intracoronary imaging in PCI increased significantly in all racial/ethnic (6.7%-13.0% [White], 6.5%-12.4% [Black], 6.9%-13.9% [Hispanic], and 8.0%-16.2% [other]; all *P*_trend_ < .01), sex (6.8%-13.7% [men] and 7.0%-12.8% [women]; both *P*_trend_ < .01), and economic groups (7.6%-16.7% [high income], 6.7%-13.3% [medium income], and 6.7%-11.5% [low income]; all *P*_trend_ < .01). Annual trends for intracoronary imaging use in PCI stratified by race/ethnicity, sex, and economic status are shown in Figure 4.

### Baseline risk profiles stratified by race/ethnicity, sex, and economic status

There were notable differences in demographic and clinical characteristics among patients undergoing intracoronary imaging across the racial/ethnic, sex, and economic groups. Black patients were younger and more likely to be women compared with other racial/ethnic groups. Women were older and more likely to be Black compared with men. Differences were also found in the economic status of each racial/ethnic and sex group, with more Black and Hispanic patients compared with White patients (50.6% and 36.8% vs 23.1%; *P* < .01) and women compared with men (29.7% vs 25.2%; *P* < .01) living in neighborhoods in the lowest quartile of median household income. Black patients, women, and low- and medium-income patients had higher Elixhauser and Charlson comorbidity index scores compared with other racial/ethnic groups, men, and high-income patients, respectively. Black patients had the highest rates of hypertension, drug abuse, obesity, congestive heart failure, renal failure requiring dialysis, and prior stroke/transient ischemic attack and Hispanic patients had the highest rates of diabetes and liver disease compared with other racial/ethnic groups. Women had higher rates of diabetes, hypertension, obesity, congestive heart failure, renal failure requiring dialysis, chronic pulmonary disease, depression, and prior stroke/transient ischemic attack compared with men. Patients with low and medium income had higher rates of diabetes, hypertension, nicotine/tobacco use, alcohol and drug abuse, peripheral arterial disease, congestive heart failure, renal failure requiring dialysis, chronic pulmonary disease, depression, and prior stroke/transient ischemic attack compared with high-income patients. Baseline characteristics among patients undergoing PCI stratified by race/ethnicity, sex, and economic status are shown in Tables 1 and 2 (with intracoronary imaging) and Supplemental Table S3 (without intracoronary imaging).

**Table 1:** Demographic and hospital characteristics of patients undergoing PCI with intracoronary imaging stratified by race/ethnicity, sex, and economic status

	Race/ethnicity					Biological sex			Economic status			
	White (n = 145,475)	Black (n = 18,365)	Hispanic (n = 16,555)	Other^a^ (n = 16,365)	*P*	Men (n = 139,030)	Women (n = 65,705)	*P*	High income (n = 45,970)	Medium income (n = 101,505)	Low income (n = 53,645)	*P*
Demographic characteristics												
Age, y	67 (58-75)	62 (53-70)	63 (55-72)	64 (55-73)	<.01	65 (56-73)	68 (59-77)	<.01	67 (58-76)	66 (57-74)	64 (56-73)	<.01
18-64	43.2	57.5	53.5	52.8	<.01	49.6	39.1	<.01	42.2	45.9	50.2	<.01
65-74	29.9	25.9	26.1	26.5		28.6	29.6		29.6	29.4	27.2	
75-84	20.4	13.3	16.5	16.1		17.0	23.1		20.6	18.8	18.2	
85+	6.5	3.3	3.9	4.7		4.7	8.2		7.6	5.9	4.5	
Race/ethnicity												
White	100	0	0	0	<.01	74.9	71.8	<.01	77.8	77.6	63.8	<.01
Black	0	100	0	0		7.9	12.3		4.6	7.1	17.7	
Hispanic	0	0	100	0		8.4	8.4		5.3	8.2	11.6	
Other	0	0	0	100		8.7	7.5		12.3	7.1	7.0	
Biological sex												
Male	68.7	57.7	67.9	70.9	<.01	100	0	<.01	71.6	68.0	64.2	<.01
Female	31.3	42.3	32.1	29.1		0	100		28.4	32.0	35.8	
Income quartile												
I	23.1	50.6	36.8	22.5	<.01	25.2	29.7	<.01	0	0	100	<.01
II	26.3	21.1	25.6	18.4		25.0	26.1		0	50.3	0	
III	26.4	17.1	23.1	24.8		25.6	24.1		0	49.7	0	
IV	24.2	11.2	14.5	34.2		24.1	20.2		100	0	0	
Insurance												
Medicare	59.0	50.9	48.4	44.2	<.01	51.9	64.3	<.01	54.9	56.0	57.3	<.01
Medicaid	7.0	17.0	18.5	17.3		9.5	10.3		6.0	8.9	14.5	
Private insurance	30.3	25.5	26.1	33.6		34.0	21.8		36.6	30.9	22.5	
Self-pay	3.7	6.6	7.0	4.8		4.6	3.6		2.6	4.2	5.7	
Hospital characteristics												
Location/teaching status												
Rural	6.0	1.9	1.6	3.1	<.01	5.2	5.2	.05	0.4	4.5	10.5	<.01
Urban nonteaching	18.4	12.0	17.2	17.4		17.8	16.8		18.0	19.7	13.1	
Urban teaching	75.5	86.2	81.1	79.4		77.0	78.0		81.6	75.8	76.4	
Bed size^b^												
Small	13.9	12.6	11.3	10.2	<.01	13.2	13.7	.06	11.6	14.5	12.8	<.01
Medium	25.7	23.8	29.9	23.3		25.4	26.0		31.9	24.9	21.6	
Large	60.4	63.6	58.8	66.4		61.5	60.2		56.5	60.6	65.7	
Region												
Northeast	17.0	15.8	21.6	33.2	<.01	18.2	18.1	<.01	33.5	15.7	10.0	<.01
Midwest	25.0	26.5	6.5	10.7		22.8	23.5		15.8	25.8	24.9	
South	33.8	48.3	34.9	22.5		33.2	35.7		19.7	32.1	49.5	
West	24.2	9.4	37.0	33.6		25.8	22.7		30.9	26.4	15.6	
Elective admission	11.8	9.1	10.9	11.7	<.01	11.4	12.0	.05	11.7	11.3	12.0	.29
Weekend admission	23.0	22.7	20.7	21.6	.02	22.9	22.0	.06	22.6	22.7	22.4	.82

Data presented as median (interquartile range) or %. Two authors (M.I. and H.A.) independently verified the International Classification of Diseases, Tenth Revision (ICD-10), codes that corresponded to each comorbidity (Supplemental Table S1), and any disagreements in inclusion or exclusion of ICD-10 codes were discussed with a third author (A.M.G).

a Other race refers to Asian or Pacific Islander, Native American, and other;

b Bed-size categories are based on inpatient beds and are specific to the hospital’s location and teaching status.

**Table 2:** Clinical characteristics of patients undergoing PCI with intracoronary imaging stratified by race/ethnicity, sex, and economic status

	Race/ethnicity					Biological sex			Economic status			
	White (n = 145,475)	Black (n = 18,365)	Hispanic (n = 16,555)	Other^a^ (n = 16,365)	*P*	Men (n = 139,030)	Women (n = 65,705)	*P*	High income (n = 45,970)	Medium income (n = 101,505)	Low income (n = 53,645)	*p*
Elixhauser comorbidity index	3 (2-5)	4 (3-5)	3 (2-5)	3 (2-5)	<.01	3 (2-5)	4 (2-6)	<.01	3 (2-5)	4 (2-5)	4 (2-5)	<.01
Charlson comorbidity index	2 (1-4)	3 (2-5)	2 (1-4)	2 (1-4)	<.01	2 (1-4)	3 (1-4)	<.01	2 (1-4)	3 (1-4)	3 (1-4)	<.01
0	5.7	3.2	4.2	7.1	<.01	5.7	4.9	<.01	7.5	5.3	4.1	<.01
1	26.6	19.9	22.5	26.3		27.6	21.6		28.8	25.8	22.6	
2	24.5	20.5	25.0	24.2		24.5	23.4		24.5	24.5	23.3	
≥3	43.2	56.5	48.3	42.4		42.2	50.1		39.2	44.5	50.0	
Diabetes mellitus	37.1	52.2	56.3	49.3	<.01	38.5	46.3	<.01	36.5	40.3	46.3	<.01
Hypertension	81.5	89.9	85.9	81.6	<.01	81.5	84.7	<.01	80.0	82.4	85.3	<.01
Dyslipidemia	75.5	70.7	74.4	75.9	<.01	75.6	73.5	<.01	77.1	75.4	72.4	<.01
Nicotine/tobacco use	52.6	51.7	42.7	42.5	<.01	53.9	44.5	<.01	45.2	51.3	54.7	<.01
Alcohol abuse	2.8	3.4	2.6	2.4	.08	3.7	1.0	<.01	2.3	2.8	3.2	<.01
Drug abuse	2.8	7.4	3.7	2.8	<.01	3.8	2.2	<.01	2.1	3.0	4.9	<.01
Obesity	22.2	26.0	21.1	16.2	<.01	20.3	25.6	<.01	19.8	22.8	22.5	<.01
Peripheral arterial disease	13.3	12.7	10.2	9.1	<.01	12.3	13.1	.03	11.6	12.4	13.9	<.01
Atrial fibrillation/flutter	18.7	11.7	11.9	12.4	<.01	17.1	16.6	.27	17.7	17.2	15.8	<.01
Congestive heart failure	40.9	46.3	42.3	37.8	<.01	40.2	43.5	<.01	37.9	41.4	43.9	<.01
Renal failure	20.2	33.1	25.2	23.0	<.01	21.1	23.8	<.01	20.5	21.8	23.7	<.01
Dialysis dependent	2.2	9.1	6.8	5.6	<.01	3.1	4.2	<.01	2.9	3.1	4.5	<.01
Liver disease	3.6	3.6	4.8	4.2	<.01	3.8	3.6	.37	3.8	3.6	4.0	.20
Chronic pulmonary disease	21.5	21.4	14.2	13.5	<.01	17.7	25.5	<.01	15.8	19.7	24.8	<.01
Obstructive sleep apnea	11.6	10.2	7.0	6.6	<.01	11.7	8.6	<.01	11.1	10.9	9.9	.02
Coagulopathy	5.1	4.9	5.7	4.9	.40	5.1	5.1	.78	5.6	4.9	5.0	.06
Cancer	2.2	1.5	1.4	1.4	<.01	2.2	1.6	<.01	2.1	2.0	2.0	.80
Malnutrition	1.2	1.9	1.3	1.5	.01	1.1	1.8	<.01	1.2	1.2	1.5	.06
Dementia	2.1	2.1	2.1	1.8	.83	1.7	2.7	<.01	2.1	2.1	2.0	.96
Depression	10.1	6.6	8.3	5.7	<.01	7.0	13.9	<.01	8.2	9.5	9.8	<.01
Previous history												
Myocardial infarction	20.2	21.0	20.0	18.3	.04	21.0	18.1	<.01	19.4	20.2	20.6	.12
Stroke/TIA	7.4	9.5	7.2	6.7	<.01	6.7	9.3	<.01	6.7	7.5	8.3	<.01
Cardiac arrest	0.8	0.9	0.6	0.6	.34	0.9	0.6	<.01	0.8	0.8	0.8	.79
PCI	21.3	22.2	19.9	19.4	.03	21.9	19.2	<.01	20.6	21.0	21.7	.26
CABG	8.2	7.3	7.8	7.2	.12	8.6	6.7	<.01	7.2	8.0	8.6	<.01
ICD	2.4	2.9	2.1	1.3	<.01	2.7	1.4	<.01	2.1	2.2	2.7	.02
PPM	3.0	1.8	2.0	1.6	<.01	2.6	2.7	.50	3.1	2.6	2.4	.01

Data presented as median (interquartile range) or %. Two authors (M.I. and H.A.) independently verified the International Classification of Diseases, Tenth Revision (ICD-10), codes that corresponded to each comorbidity (Supplemental Table S1), and any disagreements in inclusion or exclusion of ICD-10 codes were discussed with a third individual (A.M.G).

CABG, coronary artery bypass grafting; ICD, implantable cardioverter-defibrillator; PCI, percutaneous coronary intervention; PPM, permanent pacemaker; TIA, transient ischemic attack.

a Other race refers to Asian or Pacific Islander, Native American, and other.

### In-hospital outcomes stratified by race/ethnicity, sex, and economic status

Among patients undergoing intracoronary imaging, the estimated overall in-hospital mortality rate was 2.5% (95% CI, 2.3%-2.7%) and AKI rate was 16.4% (95% CI, 15.9%-16.8%). In the unadjusted analysis, Black race and low income were associated with higher unadjusted odds of AKI compared with White race and high income, respectively (both *P* < .01). Female sex was associated with higher unadjusted odds of in-hospital mortality compared with men (*P* < .01). After adjustment for potential confounders using multivariable regression analysis, Black race was associated with higher adjusted odds of AKI compared with White race (*P* < .01). The adjusted odds of AKI in low-income patients were similar compared with high-income patients (*P* = .18). Likewise, the adjusted odds of in-hospital mortality in women were similar compared with men (*P* = .56). In-hospital outcomes of intracoronary imaging stratified by race/ethnicity, sex, and economic status are summarized in Table 3.

**Table 3:** In-hospital outcomes of PCI with intracoronary imaging stratified by race/ethnicity, sex, and economic status

	Race/ethnicity					Biological sex			Economic status			
	White (n = 145,475)	Black (n = 18,365)	Hispanic (n = 16,555)	Other^a^ (n = 16,365)	*P*	Men (n = 139,030)	Women (n = 65,705)	*P*	High income (n = 45,970)	Medium income (n = 101,505)	Low income (n = 53,645)	*P*
Mortality	2.5	2.2	2.4	2.4	.77	2.2	3.1	<.01^b^	2.4	2.4	2.6	.57
uOR	Ref.	0.88 (0.70-1.11	0.99 (0.79-1.25)	0.96 (0.75-1.23	—	Ref.	1.38 (1.22-1.57)^b^	—	Ref.	1.00 (0.85-1.17)	1.07 (0.90-1.29)	—
aOR^c^	Ref.	0.90 (0.68-1.19)	0.90 (0.68-1.18)	1.03 (0.79-1.34)	—	Ref.	1.04 (0.90-1.22)	—	Ref.	1.08 (0.90-1.30)	1.20 (0.96-1.51)	—
AKI	15.8	21.6	17.0	15.6	<.01^b^	16.2	16.7	.19	15.3	16.3	17.5	<.01^b^
uOR	Ref.	1.47 (1.35-1.61)^b^	1.09 (0.98-1.21)	0.98 (0.89-1.09)	—	Ref.	1.03 (0.98-1.09)	—	Ref.	1.07 (0.99-1.16)	1.17 (1.07-1.28)^b^	—
aOR^c^	Ref.	1.40 (1.25-1.57)^b^	1.00 (0.88-1.13)	1.07 (0.95-1.21)	—	Ref.	1.01 (0.96-1.06)	—	Ref.	1.04 (0.95-1.14)	1.07 (0.96-1.19)	—

Data presented as % or OR (95% CI). The International Classification of Diseases, Tenth Revision (ICD-10) codes corresponding to acute kidney injury were identified with the same process used to identify comorbidity codes (Supplemental Table S1).

AKI, Acute kidney injury; aOR, adjusted odds ratio; uOR, unadjusted odds ratio.

a Other race refers to Asian or Pacific Islander, Native American, and other.

b Statistical significance.

c The multivariable regression model is adjusted for age, race/ethnicity (not included when comparing racial/ethnic groups), sex (not included when comparing sex groups), income (not included when comparing income groups), insurance, hospital location and teaching status, bed size, region, type of admission, Elixhauser and Charlson comorbidity index scores, and relevant comorbidities (Supplemental Table S2).

In addition to the significant differences in adjusted AKI observed among patients undergoing intracoronary imaging by race/ethnicity, patients not undergoing intracoronary imaging also exhibited significant differences in adjusted in-hospital mortality by race/ethnicity and economic status (Supplemental Table S4).

## Discussion

We report racial/ethnic, sex, and economic disparities among patients who underwent intracoronary imaging. This analysis of the large NIS database produced several novel findings (Central Illustration) that include the following: (1) among patients undergoing PCI, low and medium income were independently associated with lower intracoronary imaging use compared with high income. Black race and Hispanic ethnicity compared with White race as well as female sex were not independently associated with lower intracoronary imaging use in PCI; (2) from 2016 through 2020, the use of intracoronary imaging in PCI increased significantly in all racial/ethnic, sex, and economic groups; (3) among patients undergoing PCI with or without intracoronary imaging, Black race was associated with higher adjusted odds of AKI compared with White race; and (4) among patients undergoing PCI, in-hospital mortality disparities were ameliorated with the use of intracoronary imaging.

**Central Illustration:**
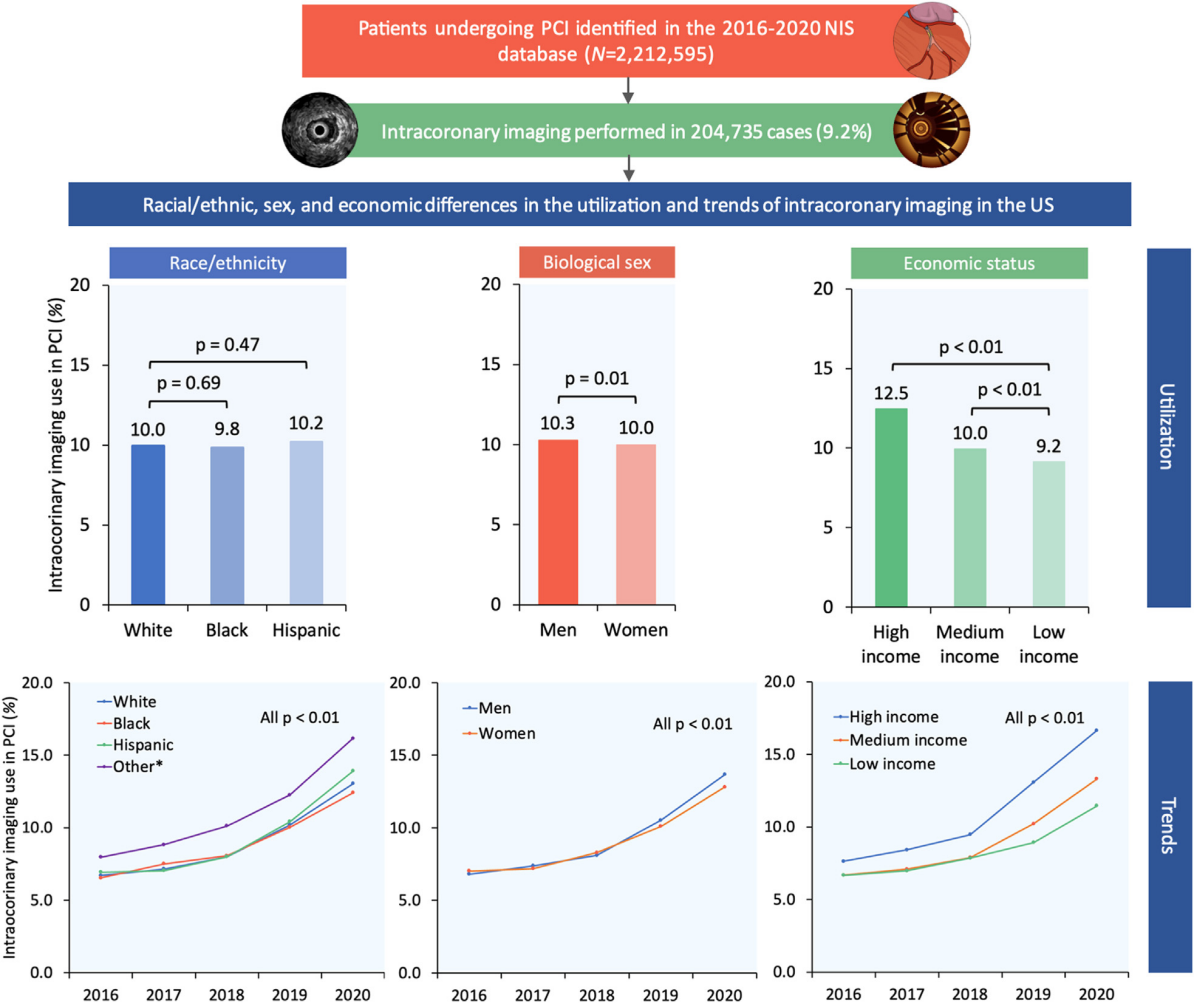
Key study findings. Reported numbers represent national-level estimates. ∗Other race refers to Asian or Pacific Islander, Native American, and other. NIS, National Inpatient Sample; PCI, percutaneous coronary intervention.

### Disparities in intracoronary imaging use in PCI

Our study found that among patients who underwent PCI, women had lower intracoronary imaging use compared with men. This was likely attributed to economic disparities between men and women because women were more likely to be of low and medium income, an independent predictor of lower intracoronary imaging use in PCI noted in our study. This is congruent with prior studies that identified low socioeconomic status as a significant barrier to coronary interventions.^15^^,^^16^ In a study by Yong et al^15^ evaluating socioeconomic inequalities in the quality of care among patients admitted with acute coronary syndrome, patients in the lowest income group were less likely to receive a coronary angiogram (aOR, 0.82; 95% CI, 0.70-0.96) and PCI (aOR, 0.82; 95% CI, 0.74-0.91) compared with patients in the highest income group.^15^ Similarly, in a meta-analysis evaluating the impact of socioeconomic status on access to cardiovascular interventions after acute myocardial infarction, low socioeconomic status was associated with lower odds of cardiac catheterization (pooled OR, 0.80; 95% CI, 0.65-0.99) and revascularization (pooled OR, 0.76; 95% CI, 0.63-0.90).^16^ In a previous survey conducted by Vemmou et al,^17^ high cost was identified as one of the most common reasons for reluctance to use intracoronary imaging by interventional cardiologists. Therefore, financial barriers in women with low and medium income may have limited their access to high-quality care and ability to afford high-cost interventions. Furthermore, possible differences in the severity and extent of coronary artery disease between men and women may have contributed to disparities in intracoronary imaging use as prior studies have shown substantially different patterns of coronary artery disease between genders, with women demonstrating lower mean extent scores (19.6 vs 36.8; *P* < .01) and lower vessel scores (0.7 vs 1.3; *P* < .01) compared with men.^18^ Finally, unconscious provider biases may have contributed to the lower utilization of intracoronary imaging in women. Given the clear benefits of intracoronary imaging in PCI, a multifaceted approach is necessary to attenuate economic disparities in the use of intracoronary imaging in PCI.

### Temporal trends in intracoronary imaging use in PCI

Our analysis showed that the use of intracoronary imaging in PCI increased over the 5-year study period in all racial/ethnic, sex, and economic groups. These findings are congruent with prior studies evaluating trends in intracoronary imaging use in PCI, both in the United States and across the globe.^19^^,^^20^ In a retrospective study evaluating trends in intracoronary imaging use in the United States*,* intracoronary imaging-guided PCI for myocardial infarction increased from 2008 to 2019 (3.4%-8.7% for IVUS-guided PCI and 0.0%-0.6% for OCT-guided PCI; both *P*_trend_ < .01).^19^ Similarly, Kim et al^20^ noted that the use of IVUS-guided PCI increased among Korean patients hospitalized with acute myocardial infarction from 15.0% in 2011 to 25.7% in 2015 (*P*_trend_ < .01). The increased prevalence of intracoronary imaging reflects the strength of current evidence and guidelines endorsing IVUS as a class 2A level of recommendation among patients undergoing PCI, with OCT as an acceptable alternative.^4^^,^^5^^,^^21^ This evidence is exemplified by a recent meta-analysis that found significantly lower risks of major adverse cardiovascular events (relative risk, 0.61; 95% CI, 0.48-0.78) and myocardial infarction (relative risk, 0.48; 95% CI, 0.25-0.95) in patients who underwent intracoronary imaging–guided PCI compared with angiography-only PCI.^22^ Although the use of intracoronary imaging in PCI has been increasing, adoption of intracoronary imaging remains poor despite an association with lower mortality.^19^

### In-hospital outcomes of PCI with and without intracoronary imaging

Our study found that among patients who underwent PCI with or without intracoronary imaging, Black race was associated with higher adjusted odds of AKI compared with White race. Black patients continue to face significant racial disparities in outcomes following PCI.^23^^,^^24^ The increased odds of AKI in Black patients align with prior studies that found that Black race was associated with increased odds of AKI following PCI compared with White race (aOR, 1.79; 95% CI, 1.48-2.15).^23^ The underlying reasons for such disparities in AKI may be related to cumulative lifetime socioeconomic status, a known predictor of adulthood kidney health among Black Americans in the Jackson Heart Study,^25^ and to poor access to health care and utilization of routine medical care, which are important for promotion of kidney health.^26^^,^^27^

Among patients who underwent PCI with intracoronary imaging, there were no significant differences in the adjusted in-hospital mortality by race/ethnicity, sex, and economic status. In contrast, patients not undergoing intracoronary imaging had significant disparities in the adjusted in-hospital mortality by race/ethnicity and economic status. These finding support that intracoronary imaging may improve care and level the playing field, resulting in improved outcomes for all patients.

### Limitations

Our study has several important limitations. First, racial/ethnic demographic characteristics are often self-described, self-reported, or entered by a clerk. Hence, the chances of errors in allocation of patients to different racial/ethnic categories cannot be excluded. Second, in a retrospective NIS study using administrative claims codes, incorrect coding could lead to inaccurate data. Third, the retrospective nature of the study makes it subject to inherent selection bias. Fourth, detailed baseline and procedural characteristics such as access site, contrast volume, coronary anatomy, site of PCI, and periprocedural medications were unavailable, which can lead to unmeasured bias. Fifth, validated grading scores such as the SYNTAX score are not captured by the NIS, limiting patient risk assessment. Finally, NIS allows detailed assessment of in-hospital outcomes but does not include long-term clinical outcomes beyond discharge. Studies exploring the long-term racial/ethnic, sex, and economic disparities in the outcomes of intracoronary imaging in PCI are still needed.

Despite these limitations, the study adds meaningfully to the literature by describing contemporary racial/ethnic, sex, and economic disparities in the utilization and outcomes of intracoronary imaging in PCI. The NIS is well validated for outcomes studies like this one and undergoes serial data accuracy checks and quality control. In addition, the NIS data are geographically diverse, including a nationally representative sample of centers and operators, and hence reliably reflect real-world practice and outcomes.

## Conclusions

Among patients undergoing PCI, low and medium income were independently associated with lower intracoronary imaging use compared with high income. Black race and Hispanic ethnicity compared with White race as well as female sex were not independently associated with lower intracoronary imaging use in PCI. Further studies are needed to identify effective strategies to mitigate economic disparities in intracoronary imaging use and provide health equity for all patients.
