# Supplementary material for: Racial/Ethnic, Sex, and Economic Disparities in the Utilization and Outcomes of Intracoronary Imaging
Source: J Soc Cardiovasc Angiogr Interv. 2024 May 11;3(6):101936. doi: 10.1016/j.jscai.2024.101936 (PMC11308510; doi:10.1016/j.jscai.2024.101936)
Supplement: Supplemental Material [file mmc1.docx]

**Supplemental Material**

**Table S1.** ICD-10 diagnosis (CM) and procedure (PCS) codes

| **Procedure** | **ICD-10 PCS codes** |
| --- | --- |
| Percutaneous coronary intervention (PCI) | 02703ZZ, 027034Z, 0270346, 0270356, 027035Z, 0270366, 027036Z, 0270376, 027037Z, 02703Z6, 0270446, 027044Z, 0270456, 027045Z, 0270466, 027046Z, 0270476, 027047Z, 02704Z6, 02704ZZ, 0271356, 027135Z, 0271366, 027136Z, 0271376, 027137Z, 02713Z6, 02713ZZ, 0271446, 027144Z, 0271456, 027145Z, 0271466, 027146Z, 0271476, 027147Z, 02714Z6, 02714ZZ, 0272346, 027234Z, 0272356, 027235Z, 0272366, 027236Z, 0272376, 027237Z, 02723Z6, 02723ZZ, 0272446, 027244Z, 0272456, 027245Z, 0272466, 027246Z, 0272476, 027247Z, 02724Z6, 02724ZZ, 0273346, 027334Z, 0273356, 027335Z, 0273366, 027336Z, 0273376, 027337Z, 02733Z6, 02733ZZ, 0273446, 027344Z, 0273456, 027345Z, 0273466, 027346Z, 0273476, 027347Z, 02734Z6, 02734ZZ, 02C00ZZ, 02C10ZZ, 02C20ZZ, 02C30ZZ, 3E07317, 3E073PZ, 02C03ZZ, 02C04ZZ, 02C13ZZ, 02C14ZZ, 02C23ZZ, 02C24ZZ, 02C33ZZ, 02C34ZZ |
| Intravascular ultrasound (IVUS) | B240ZZ3, B241ZZ3, B244ZZ3, B245ZZ3, B246ZZ3, B24DZZ3 |
| Optical coherence tomography (OCT) | B221Z2Z, B223Z2Z |

| **Baseline characteristics** | **ICD-10 CM codes** |
| --- | --- |
| **Comorbidities** | |
| Diabetes mellitus | E10.0, E10.1, E10.9, E11.0, E11.1, E11.9, E12.0, E12.1, E12.9, E13.0, E13.1, E13.9, E14.0, E14.1, E14.9, E10.2-E10.8, E11.2-E11.8, E12.2-E12.8, E13.2-E13.8, E14.2-E14.8 |
| Hypertension | I10.x, I11.x-I13.x, I15.x |
| Dyslipidemia | E78.x |
| Nicotine/tobacco use | F17.x, Z72.0, Z87.891 |
| Alcohol abuse | F10, E52, G62.1, I42.6, K29.2, K70.0, K70.3, K70.9, T51.x, Z50.2, Z71.4, Z72.1 |
| Drug abuse | F11.x-F16.x, F18.x, F19.x, Z71.5. Z72.2 |
| Obesity | E66.x |
| Peripheral vascular disease | I70.x, I71.x, I73.1, I73.8, I73.9, I77.1, I79.0, I79.2, K55.1, K55.8, K55.9, Z95.8, Z95.9 |
| Atrial fibrillation/flutter | I48.x |
| Congestive heart failure | I09.9, I11.0, I13.0, I13.2, I25.5, I42.0, 142.5-I42.9, I43.x, I50.x, P29.0 |
| Renal failure | I12.0, I13.1, N18.x, N19.x, N25.0, Z49.0-Z49.2, Z94.0, Z199.2 |
| Dialysis dependent | Z99.2 |
| Liver disease | B18.x, I85.x, I86.4, I98.2, K70.x, K71.1, K71.3-K71.5, K71.7, K72.x-K74.x, K76.0, K76.2-K76.9. Z94.4 |
| Chronic pulmonary disease | I27.8, 127.9, J40.x-J47.x, J60.x-J67.x, J68.4, J70.1, J70.3 |
| Obstructive sleep apnea | G47.33 |
| Coagulopathy | D65-D68.x, D69.1, D69.3-D69.6 |
| Cancer | C0x.x, C1x.x, C2x.x, C30.x, C31.x, C32.x, C33.x, C34.x, C37.x, C38.x, C39.x, C40.x, C41.x, C43.x, C45.x, C46.x, C47.x, C48.x, C49.x, C50, C51-58.x, C60-63.x, C76.x, C80.1, C81.x, C82.x, C83.x, C84.x, C85.x, C88.x, C9x.x |
| Malnutrition | E43, E44.x, E45, E46 |
| Dementia | F01.x, F02.x, F03.x, F04, F05, F06.1, F06.8, G13.2, G13.8, G30.x, G31.0x, G31.1, G31.2, G91.4, G94, R41.81, R54 |
| Depression | F20.4, F31.3-F31.5, F32.x, F33.x, F34.1, F41.2, F43.2 |
| **Previous history** | |
| Myocardial infarction | I25.2 |
| Stroke/TIA | Z86.73 |
| Cardiac arrest | Z86.74 |
| PCI | Z98.61, Z95.5 |
| CABG | Z95.1 |
| ICD | Z95.810 |
| PPM | Z95.0 |

| **In-hospital outcomes** | **ICD-10 CM codes** |
| --- | --- |
| Acute kidney injury | N17.-, N99.0 |

**Table S2.** Variables used in the multivariable regression analysis to compute adjusted odds of in-hospital outcomes

| **Demographic characteristics** |
| --- |
| Age |
| Race/ethnicity (not included when comparing racial/ethnic groups) |
| Biological sex (not included when comparing sex groups) |
| Income quartile (not included when comparing income groups) |
| Insurance |
| **Hospital characteristics** |
| Location/teaching status |
| Bed size |
| Region |
| Elective admission |
| Weekend admission |
| **Comorbidities** |
| Elixhauser comorbidity index |
| Charlson comorbidity index |
| Diabetes mellitus |
| Hypertension |
| Dyslipidemia |
| Nicotine/tobacco use |
| Alcohol abuse |
| Drug abuse |
| Obesity |
| Peripheral arterial disease |
| Atrial fibrillation/atrial flutter |
| Congestive heart failure |
| Renal failure |
| Dialysis dependent |
| Liver disease |
| Chronic pulmonary disease |
| Obstructive sleep apnea |
| Coagulopathy |
| Cancer |
| Malnutrition |
| Dementia |
| Depression |
| **Previous** **history** |
| Myocardial infarction |
| Stroke/TIA |
| Cardiac arrest |
| PCI |
| CABG |
| ICD |
| PPM |

**Table S3.** Baseline characteristics of patients undergoing PCI without intracoronary imaging stratified by race/ethnicity, sex, and economic status

|  | **Race/ethnicity** | | | | | **Biological sex** | | | **Economic status** | | | |
| --- | --- | --- | --- | --- | --- | --- | --- | --- | --- | --- | --- | --- |
|  | White  (*n*= 1,460,640) | Black  (*n*=186,770) | Hispanic  (*n*=161,990) | Other*  (*n*=130,945) | *p* | Men  (*n*=1,350,715) | Women  (*n*=657,145) | *p* | High income  (*n*=368,690) | Medium income  (*n*=1,019,025) | Low income  (*n*=585,690) | *p* |
| **Demographic characteristics** |  |  |  |  |  |  |  |  |  |  |  |  |
| Age | 67 (58-75) | 62 (53-70) | 63 (55-72) | 64 (55-72) | <0.01 | 64 (56-73) | 68 (59-77) | <0.01 | 67 (57-75) | 66 (57-75) | 64 (56-73) | <0.01 |
| 18-64 | 44.1 | 59.0 | 54.1 | 52.0 | <0.01 | 50.8 | 39.1 | <0.01 | 44.2 | 46.2 | 50.0 | <0.01 |
| 65-74 | 29.2 | 25.8 | 26.1 | 26.9 |  | 28.1 | 29.2 |  | 28.6 | 28.6 | 28.0 |  |
| 75-84 | 19.9 | 12.3 | 15.4 | 16.6 |  | 16.4 | 22.9 |  | 19.8 | 18.9 | 17.1 |  |
| 85+ | 6.8 | 2.9 | 4.4 | 4.5 |  | 4.7 | 8.8 |  | 7.4 | 6.3 | 5.0 |  |
| Race/ethnicity |  |  |  |  |  |  |  |  |  |  |  |  |
| White | 100 | 0 | 0 | 0 | <0.01 | 76.2 | 73.4 | <0.01 | 77.7 | 79.3 | 67.1 | <0.01 |
| Black | 0 | 100 | 0 | 0 |  | 8.1 | 12.7 |  | 4.7 | 7.0 | 17.2 |  |
| Hispanic | 0 | 0 | 100 | 0 |  | 8.5 | 8.0 |  | 6.1 | 7.7 | 10.7 |  |
| Other | 0 | 0 | 0 | 100 |  | 7.2 | 5.9 |  | 11.5 | 6.0 | 5.0 |  |
| Biological sex |  |  |  |  |  |  |  |  |  |  |  |  |
| Male | 68.0 | 56.8 | 68.5 | 71.2 | <0.01 | 100 | 0 | <0.01 | 71.9 | 67.4 | 63.9 | <0.01 |
| Female | 32.0 | 43.2 | 31.5 | 28.8 |  | 0 | 100 |  | 28.1 | 32.6 | 36.1 |  |
| Income quartile |  |  |  |  |  |  |  |  |  |  |  |  |
| I | 26.5 | 53.3 | 38.3 | 22.1 | <0.01 | 28.2 | 32.7 | <0.01 | 0 | 0 | 100 | <0.01 |
| II | 29.0 | 21.9 | 26.2 | 20.7 |  | 27.4 | 28.3 |  | 0 | 53.6 | 0 |  |
| III | 25.1 | 15.6 | 21.8 | 25.1 |  | 24.4 | 23.1 |  | 0 | 46.4 | 0 |  |
| IV | 19.3 | 9.2 | 13.7 | 32.1 |  | 20.0 | 16.0 |  | 100 | 0 | 0 |  |
| Insurance |  |  |  |  |  |  |  |  |  |  |  |  |
| Medicare | 58.1 | 50.5 | 47.5 | 44.0 | <0.01 | 51.0 | 64.3 | <0.01 | 52.8 | 55.8 | 56.6 | <0.01 |
| Medicaid | 7.3 | 16.7 | 16.7 | 17.6 |  | 9.4 | 10.2 |  | 6.2 | 9.0 | 12.9 |  |
| Private insurance | 30.5 | 25.7 | 27.2 | 32.7 |  | 34.2 | 21.8 |  | 38.0 | 30.6 | 24.1 |  |
| Self-pay | 4.1 | 7.1 | 8.6 | 5.7 |  | 5.4 | 3.7 |  | 3.0 | 4.6 | 6.4 |  |
| **Hospital characteristics** |  |  |  |  |  |  |  |  |  |  |  |  |
| Location/teaching status |  |  |  |  |  |  |  |  |  |  |  |  |
| Rural | 6.7 | 3.8 | 1.1 | 1.9 | <0.01 | 5.5 | 6.1 | <0.01 | 0.4 | 4.6 | 10.8 | <0.01 |
| Urban nonteaching | 21.5 | 16.0 | 21.1 | 19.4 |  | 20.8 | 20.8 |  | 20.5 | 22.2 | 18.5 |  |
| Urban teaching | 71.9 | 80.2 | 77.8 | 78.7 |  | 73.7 | 73.1 |  | 79.0 | 73.2 | 70.6 |  |
| Bed size |  |  |  |  |  |  |  |  |  |  |  |  |
| Small | 16.0 | 15.0 | 14.6 | 14.8 | <0.01 | 15.7 | 15.9 | 0.07 | 17.2 | 16.5 | 13.7 | <0.01 |
| Medium | 29.5 | 31.1 | 34.1 | 28.3 |  | 29.8 | 30.0 |  | 31.6 | 29.4 | 29.4 |  |
| Large | 54.5 | 53.9 | 51.2 | 56.8 |  | 54.5 | 54.1 |  | 51.1 | 54.1 | 56.9 |  |
| Region |  |  |  |  |  |  |  |  |  |  |  |  |
| Northeast | 17.0 | 14.9 | 15.4 | 26.9 | <0.01 | 17.4 | 16.4 | <0.01 | 29.9 | 16.6 | 9.9 | <0.01 |
| Midwest | 26.3 | 19.2 | 6.7 | 11.0 |  | 23.3 | 24.1 |  | 18.6 | 27.4 | 20.8 |  |
| South | 41.5 | 57.7 | 45.0 | 28.8 |  | 41.2 | 43.5 |  | 26.1 | 38.4 | 57.9 |  |
| West | 15.3 | 8.2 | 32.8 | 33.3 |  | 18.1 | 16.0 |  | 25.4 | 17.6 | 11.4 |  |
| Elective admission | 9.2 | 7.4 | 9.4 | 10.1 | <0.01 | 9.1 | 9.4 | <0.01 | 9.2 | 9.1 | 9.4 | 0.20 |
| Weekend admission | 24.0 | 24.2 | 23.9 | 23.0 | <0.01 | 24.0 | 23.7 | <0.01 | 23.3 | 24.0 | 24.2 | <0.01 |
| **Clinical characteristics** |  |  |  |  |  |  |  |  |  |  |  |  |
| Elixhauser comorbidity index | 3 (2-5) | 4 (2-5) | 3 (2-5) | 3 (2-5) | <0.01 | 3 (2-5) | 4 (2-5) | <0.01 | 3 (2-5) | 4 (2-5) | 4 (2-5) | <0.01 |
| Charlson comorbidity index | 2 (1-4) | 3 (2-5) | 2 (1-4) | 2 (1-4) | <0.01 | 2 (1-4) | 3 (1-4) | <0.01 | 2 (1-3) | 2 (1-4) | 2 (1-4) | <0.01 |
| 0 | 4.7 | 3.1 | 3.7 | 5.2 | <0.01 | 4.7 | 4.1 | <0.01 | 5.8 | 4.5 | 3.7 | <0.01 |
| 1 | 28.4 | 21.4 | 24.3 | 27.6 |  | 29.7 | 22.8 |  | 31.1 | 27.8 | 24.6 |  |
| 2 | 25.4 | 23.2 | 26.0 | 26.3 |  | 25.5 | 25.0 |  | 25.3 | 25.5 | 25.2 |  |
| ≥3 | 41.5 | 52.3 | 45.9 | 40.9 |  | 40.0 | 48.1 |  | 37.8 | 42.3 | 46.6 |  |
| Diabetes mellitus | 38.1 | 50.6 | 56.0 | 50.1 | <0.01 | 39.1 | 46.4 | <0.01 | 36.5 | 41.0 | 45.6 | <0.01 |
| Hypertension | 80.8 | 89.4 | 84.4 | 82.1 | <0.01 | 80.5 | 84.6 | <0.01 | 79.3 | 81.4 | 84.2 | <0.01 |
| Dyslipidemia | 73.4 | 68.9 | 72.3 | 73.4 | <0.01 | 73.2 | 72.1 | <0.01 | 74.8 | 73.4 | 70.9 | <0.01 |
| Nicotine/tobacco use | 52.9 | 52.2 | 41.5 | 41.0 | <0.01 | 54.0 | 45.1 | <0.01 | 44.4 | 51.4 | 54.8 | <0.01 |
| Alcohol abuse | 2.9 | 3.7 | 3.0 | 2.1 | <0.01 | 3.8 | 1.0 | <0.01 | 2.5 | 2.8 | 3.3 | <0.01 |
| Drug abuse | 2.8 | 8.0 | 3.5 | 2.3 | <0.01 | 3.8 | 2.4 | <0.01 | 2.1 | 3.0 | 4.6 | <0.01 |
| Obesity | 21.8 | 23.8 | 21.7 | 15.3 | <0.01 | 20.1 | 24.2 | <0.01 | 19.5 | 21.9 | 22.0 | <0.01 |
| Peripheral arterial disease | 11.7 | 11.8 | 9.7 | 8.9 | <0.01 | 11.2 | 11.6 | <0.01 | 10.5 | 11.2 | 12.0 | <0.01 |
| Atrial fibrillation/flutter | 17.4 | 10.4 | 11.6 | 12.1 | <0.01 | 15.8 | 15.8 | 0.80 | 16.4 | 16.1 | 15.0 | <0.01 |
| Congestive heart failure | 36.6 | 42.4 | 38.0 | 35.8 | <0.01 | 36.1 | 39.5 | <0.01 | 34.3 | 37.0 | 39.3 | <0.01 |
| Renal failure | 18.7 | 29.8 | 23.4 | 21.3 | <0.01 | 19.5 | 21.6 | <0.01 | 18.9 | 20.0 | 21.5 | <0.01 |
| Dialysis dependent | 1.8 | 7.6 | 6.0 | 4.9 | <0.01 | 2.6 | 3.5 | <0.01 | 2.4 | 2.7 | 3.6 | <0.01 |
| Liver disease | 3.2 | 3.7 | 4.4 | 4.0 | <0.01 | 3.4 | 3.2 | <0.01 | 3.4 | 3.3 | 3.4 | 0.28 |
| Chronic pulmonary disease | 20.9 | 19.5 | 13.6 | 13.7 | <0.01 | 17.0 | 24.7 | <0.01 | 14.9 | 19.2 | 23.1 | <0.01 |
| Obstructive sleep apnea | 10.0 | 8.4 | 6.1 | 5.7 | <0.01 | 10.2 | 7.2 | <0.01 | 9.8 | 9.6 | 8.4 | <0.01 |
| Coagulopathy | 4.5 | 4.3 | 4.9 | 5.1 | <0.01 | 4.7 | 4.2 | <0.01 | 4.9 | 4.5 | 4.4 | <0.01 |
| Cancer | 2.0 | 1.8 | 1.3 | 1.3 | <0.01 | 2.0 | 1.7 | <0.01 | 2.2 | 1.9 | 1.7 | <0.01 |
| Malnutrition | 1.1 | 1.5 | 1.4 | 1.3 | <0.01 | 1.0 | 1.5 | <0.01 | 1.1 | 1.1 | 1.3 | <0.01 |
| Dementia | 2.4 | 2.4 | 2.4 | 1.8 | <0.01 | 1.9 | 3.2 | <0.01 | 2.2 | 2.3 | 2.5 | <0.01 |
| Depression | 9.8 | 6.7 | 6.9 | 5.1 | <0.01 | 6.7 | 13.4 | <0.01 | 7.9 | 9.0 | 9.4 | <0.01 |
| Previous history |  |  |  |  |  |  |  |  |  |  |  |  |
| Myocardial infarction | 18.4 | 18.7 | 17.0 | 16.2 | <0.01 | 18.8 | 16.4 | <0.01 | 17.0 | 18.0 | 18.8 | <0.01 |
| Stroke/TIA | 7.1 | 9.2 | 6.9 | 5.7 | <0.01 | 6.4 | 8.8 | <0.01 | 6.1 | 7.1 | 7.9 | <0.01 |
| Cardiac arrest | 0.6 | 0.6 | 0.4 | 0.5 | <0.01 | 0.6 | 0.5 | <0.01 | 0.6 | 0.6 | 0.5 | <0.01 |
| PCI | 20.5 | 20.0 | 18.7 | 19.0 | <0.01 | 20.9 | 18.5 | <0.01 | 19.2 | 20.0 | 20.9 | <0.01 |
| CABG | 10.4 | 8.0 | 9.2 | 9.0 | <0.01 | 11.0 | 7.8 | <0.01 | 8.9 | 10.0 | 10.6 | <0.01 |
| ICD | 2.0 | 2.7 | 1.8 | 1.4 | <0.01 | 2.3 | 1.2 | <0.01 | 1.7 | 1.9 | 2.2 | <0.01 |
| PPM | 2.8 | 1.7 | 2.0 | 2.1 | <0.01 | 2.5 | 2.7 | <0.01 | 2.5 | 2.6 | 2.5 | <0.01 |

Data presented as median (IQR) or %.

*Other race refers to Asian or Pacific Islander, Native American, and Other.

**Table S4.** In-hospital outcomes of PCI without intracoronary imaging stratified by race/ethnicity, sex, and economic status

|  | **Race/ethnicity** | | | | | **Biological sex** | | | **Economic status** | | | |
| --- | --- | --- | --- | --- | --- | --- | --- | --- | --- | --- | --- | --- |
|  | White  (*n*= 1,460,640) | Black  (*n*=186,770) | Hispanic  (*n*=161,990) | Other*  (*n*=130,945) | *p* | Men  (*n*=1,350,715) | Women  (*n*=657,145) | *p* | High income  (*n*=368,690) | Medium income  (*n*=1,019,025) | Low income  (*n*=585,690) | *p* |
| **Mortality** | 2.9 | 2.6 | 3.1 | 3.4 | **<0.01** | 2.7 | 3.5 | **<0.01** | 2.9 | 2.9 | 3.1 | **0.01** |
| uOR (95% CI) | Ref. | **0.90 (0.84-0.96)** | **1.10 (1.03-1.17)** | **1.19 (1.10-1.28)** | - | Ref. | **1.30 (1.25-1.35)** | - | Ref. | 1.00 (0.95-1.06) | **1.07 (1.01-1.13)** | - |
| aOR (95% CI)^†^ | Ref. | **0.89 (0.82-0.96)** | 0.99 (0.92-1.07) | **1.16 (1.07-1.26)** | - | Ref. | 0.97 (0.92-1.01) | - | Ref. | **1.06 (1.01-1.14)** | **1.18 (1.10-1.27)** | - |
| **Acute kidney injury** | 14.8 | 19.5 | 17.7 | 15.8 | **<0.01** | 15.4 | 15.7 | 0.12 | 15.0 | 15.2 | 16.3 | **<0.01** |
| uOR (95% CI) | Ref. | **1.4 (1.36-1.44)** | **1.23 (1.19-1.28)** | **1.08 (1.04-1.13)** | - | Ref. | 1.02 (0.98-1.06) | - | Ref. | 1.02 (0.99-1.05) | **1.10 (1.07-1.14)** | - |
| aOR (95% CI)^†^ | Ref. | **1.31 (1.26-1.36)** | **1.22 (1.17-1.27)** | **1.13 (1.08-1.18)** | - | Ref. | 1.01 (0.99-1.03) | - | Ref. | 0.98 (0.95-1.01) | 1.01 (0.98-1.05) | - |

Data presented as % or OR (95% CI). The bold values indicate statistical significance.

*Other race refers to Asian or Pacific Islander, Native American, and Other.

^†^The multivariable regression model is adjusted for age, race/ethnicity (not included when comparing racial/ethnic groups), sex (not included when comparing sex groups), income (not included when comparing income groups), insurance, hospital location and teaching status, bed size, region, type of admission, Elixhauser and Charlson comorbidity index scores, and relevant comorbidities (*Table S2*).
